# Trends and collaborations in discoid lupus erythematosus research: a bibliometric analysis from 2010 to 2024

**DOI:** 10.3389/fmed.2025.1556976

**Published:** 2025-06-20

**Authors:** Daichao Zhang, Chaoran Liang, Qiuyue Yin, Yatong Li

**Affiliations:** ^1^Department of Public Health, Qinghai University Medical College, Xining, China; ^2^School and Hospital of Stomatology, Cheeloo College of Medicine, Shandong University & Shandong Key Laboratory of Oral Tissue Regeneration & Shandong Engineering Research Center of Dental Materials and Oral Tissue Regeneration & Shandong Provincial Clinical Research Center for Oral Diseases, Jinan, China

**Keywords:** discoid lupus erythematosus, bibliometric analysis, collaboration networks, diagnostic criteria, immunology

## Abstract

**Introduction:**

Discoid lupus erythematosus (DLE) is a chronic autoimmune skin disorder. Research fragmentation in DLE limits cohesive clinical and scientific progress. This bibliometric analysis aimed to clarify publication trends, collaboration networks, and emergent research themes in DLE from 2010 to 2024.

**Methods:**

A comprehensive search of the Web of Science Core Collection (WoSCC) used the terms “discoid lupus erythematosus” OR “lupus erythematosus discoid.” English-language articles and reviews (*n* = 861) were identified and analyzed via the Bibliometrix package in R to examine annual output, authorship, core journals, and keywords evolution.

**Results:**

Annual publications increased notably after 2018, although average citation rates declined. A small group of prolific authors, led by WERTH VP, contributed disproportionately. The United States dominated publication volume and international collaboration, followed by Italy, the United Kingdom, Germany, and China. Keywords analysis showed a shift from initial emphasis on disease classification and diagnosis toward advanced therapies, immunological mechanisms.

**Conclusion:**

Despite growing interest in DLE, it remains underrepresented compared with systemic lupus erythematosus. Broader collaborations, refined diagnostic criteria, and robust clinical trials are essential to enhance therapeutic strategies and patient outcomes in DLE.

## 1 Introduction

Discoid lupus erythematosus (DLE) is a chronic autoimmune skin disorder that predominantly affects sun-exposed areas, such as the face, ears, and scalp (1, 2). It is clinically characterized by erythematous, scaly plaques, which, if left untreated, may progress to scarring, atrophy, and dyspigmentation (3). Although DLE can occur as an isolated condition, it is also recognized as a cutaneous manifestation of systemic lupus erythematosus (SLE), a complex autoimmune disease that can affect multiple organ systems (4, 5).

The epidemiology of DLE is influenced by a combination of genetic, environmental, and immunological factors. While DLE is observed in all racial and ethnic groups, studies suggest a higher prevalence in African American populations compared to Caucasians. Additionally, the disease disproportionately affects women, with a female-to-male ratio of approximately 3:1 (6, 7). The pathogenesis of DLE is multifactorial, involving genetic predisposition, ultraviolet radiation exposure, and other environmental triggers (8, 9). Recent studies have also highlighted the role of epigenetic modifications in the development of DLE, adding a layer of complexity to its pathophysiology (10). This intricate etiology underscores the need for a comprehensive and individualized approach to the management and treatment of DLE.

Hydroxychloroquine has long been established as the cornerstone of DLE therapy, demonstrating efficacy in reducing disease activity and preventing disease progression (11, 12). In recent years, advances in understanding the underlying pathophysiological mechanisms of DLE have paved the way for exploring novel therapeutic strategies (13). Emerging biologic agents, such as belimumab, which have shown effectiveness in treating SLE, are currently being investigated for their potential application in DLE management (14, 15). However, the majority of clinical trials in lupus research focus on SLE, leaving DLE underrepresented in the literature. This research gap highlights the need for greater focus on DLE in future studies to improve outcomes for individuals affected by this condition.

Bibliometric analysis is a valuable tool for systematically evaluating trends, collaborations, and emerging research hotspots within a specific domain (16). By quantitatively analyzing large volumes of scholarly literature, bibliometric methods offer insights into the developmental trajectory of a field, identify influential publications and authors, and uncover underexplored areas of research (17). Moreover, bibliometric analysis can accelerate therapeutic innovation by revealing key studies and emerging research trends. By identifying influential clinical trials and mechanistic research, it can uncover overlooked therapeutic targets or drug classes, guiding resource allocation toward promising interventions (18). Additionally, bibliometric insights promote interdisciplinary collaboration and help researchers focus on high-impact studies addressing clinical gaps (19). This approach is particularly useful for DLE research, given its multidisciplinary nature and the fragmented distribution of studies across dermatology, immunology, and rheumatology journals. Tools such as CiteSpace, VOSviewer, and Bibliometrix allow researchers to construct co-authorship networks, visualize keywords co-occurrence, and analyze citation patterns (20, 21). These methods can help map the research landscape of DLE and provide a foundation for future investigations.

Despite the growing interest in DLE, the literature remains fragmented, with limited integration across different research domains. Moreover, there is a lack of comprehensive bibliometric analyses that synthesize current knowledge and identify emerging trends in DLE research. Such analyses are crucial for guiding future studies, optimizing resource allocation, and promoting international collaboration (22).

This study aims to address this gap by conducting a comprehensive bibliometric analysis of DLE-related literature, focusing on publication trends, influential authors, co-authorship and collaboration networks, keywords co-occurrence, major research themes, and emerging trends in the field. The goal is to provide a systematic and intuitive overview of the DLE research landscape, serving as a valuable resource for clinicians, researchers, and policymakers. The findings will not only enhance our understanding of the field but also inform the development of targeted strategies to improve patient outcomes.

## 2 Methods

### 2.1 Data sources and search strategies

In this study, a bibliometric analysis of the literature on DLE was conducted using data retrieved from the Web of Science Core Collection (WoSCC) database (23). It is a multidisciplinary citation database that provides comprehensive citation metadata. By contrast, PubMed focuses on biomedical literature and includes preprints, while Google Scholar aggregates a broader array of sources, including gray literature and non-English publications, but lacks consistent metadata (24). WoSCC was chosen for its detailed citation network capabilities, and suitability for evaluating trends in high-impact, peer-reviewed research, although this may exclude some emerging preprints or region-specific journals (25).

**Figure 1:**
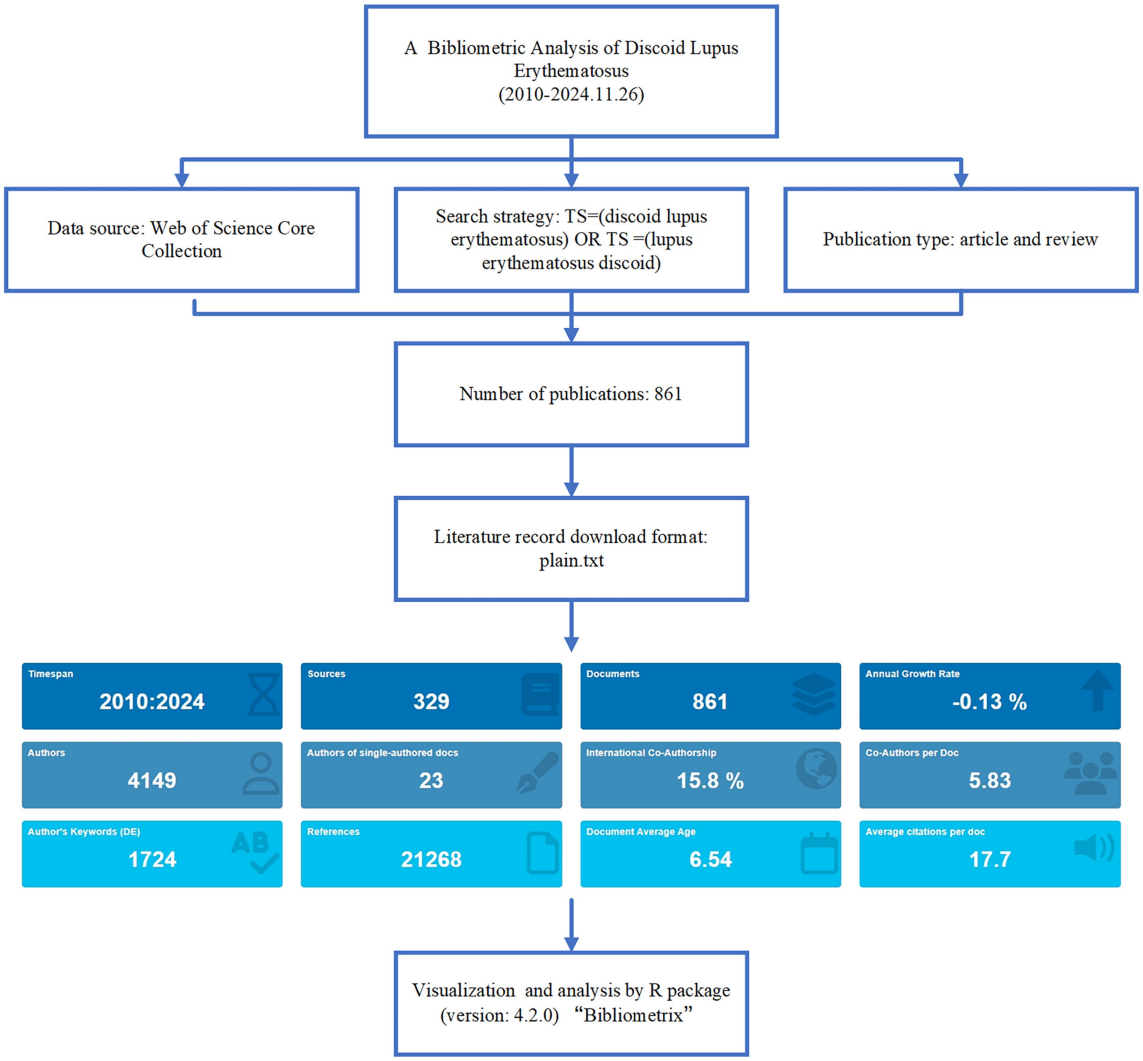
Flowchart for the bibliometric analysis of discoid lupus erythematosus.

The search was performed on November 26, 2024, covering the period from January 1, 2010, to November 26, 2024. The search strategy employed was as follows: TS = (“discoid lupus erythematosus”) OR TS = (“lupus erythematosus discoid”). The document types included articles and reviews, with non-relevant literature, conference proceedings, and non-research outputs excluded. Only documents published in English were considered. The search results were exported in txt format, containing complete metadata for further analysis (Figure 1).

### 2.2 Bibliometric analysis

The retrieved dataset was processed and analyzed using the Bibliometrix package (version 4.2.3) in R (26). This software was utilized to conduct various bibliometric analyses, enabling a comprehensive exploration of trends and patterns within the DLE research literature.

The initial bibliometric analysis was conducted using the “biblioAnalysis()” and “summary()” functions from the Bibliometrix package. These functions provided key metrics, including the total number of publications, citation counts, and the distribution of publications over time. The “biblioAnalysis()” function enabled the identification of leading authors, journals, and countries contributing to the field, while the “summary()” function summarized key indicators. To examine research collaborations, the “metaTagExtraction()” and “Biblionetwork()” functions were employed to identify collaboration patterns among countries, and authors. The results were visually represented using the “NetworkPlot()” function, which generated network maps displaying relationships based on co-authorship and co-citation patterns. The analysis also incorporated a keywords co-occurrence network, offering insights into the thematic evolution of DLE research. This was achieved through the “Biblioshiny()” function, which provides an interactive platform for visualizing bibliometric data. Additionally, thematic maps and co-occurrence networks were generated, facilitating a detailed exploration of emerging research hotspots within the field of DLE.

## 3 Results

### 3.1 Annual distribution

**Figure 2:**
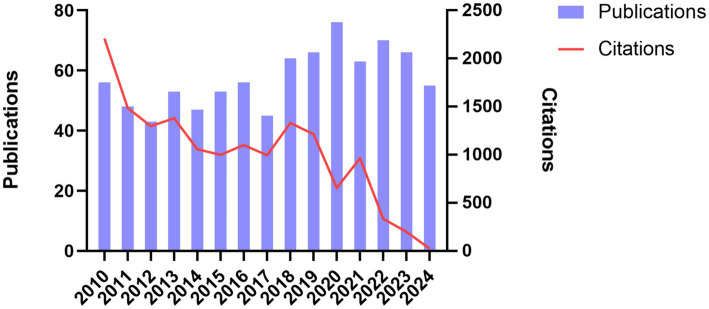
Annual distribution of publications and total citations on discoid lupus erythematosus.

A total of 861 publications on DLE were retrieved from the WoSCC database, comprising both articles and reviews. From 2010 to 2017, the annual publication volume fluctuated, with fewer than 60 publications per year. However, from 2018 to 2024, the number of publications increased, with an average of more than 60 articles per year. Despite the rise in publication volume, the total number of citations showed a downward trend from 2010 to 2024 (Figure 2), gradually dropping from over 2,000.

### 3.2 Three-factor analysis

**Figure 3:**
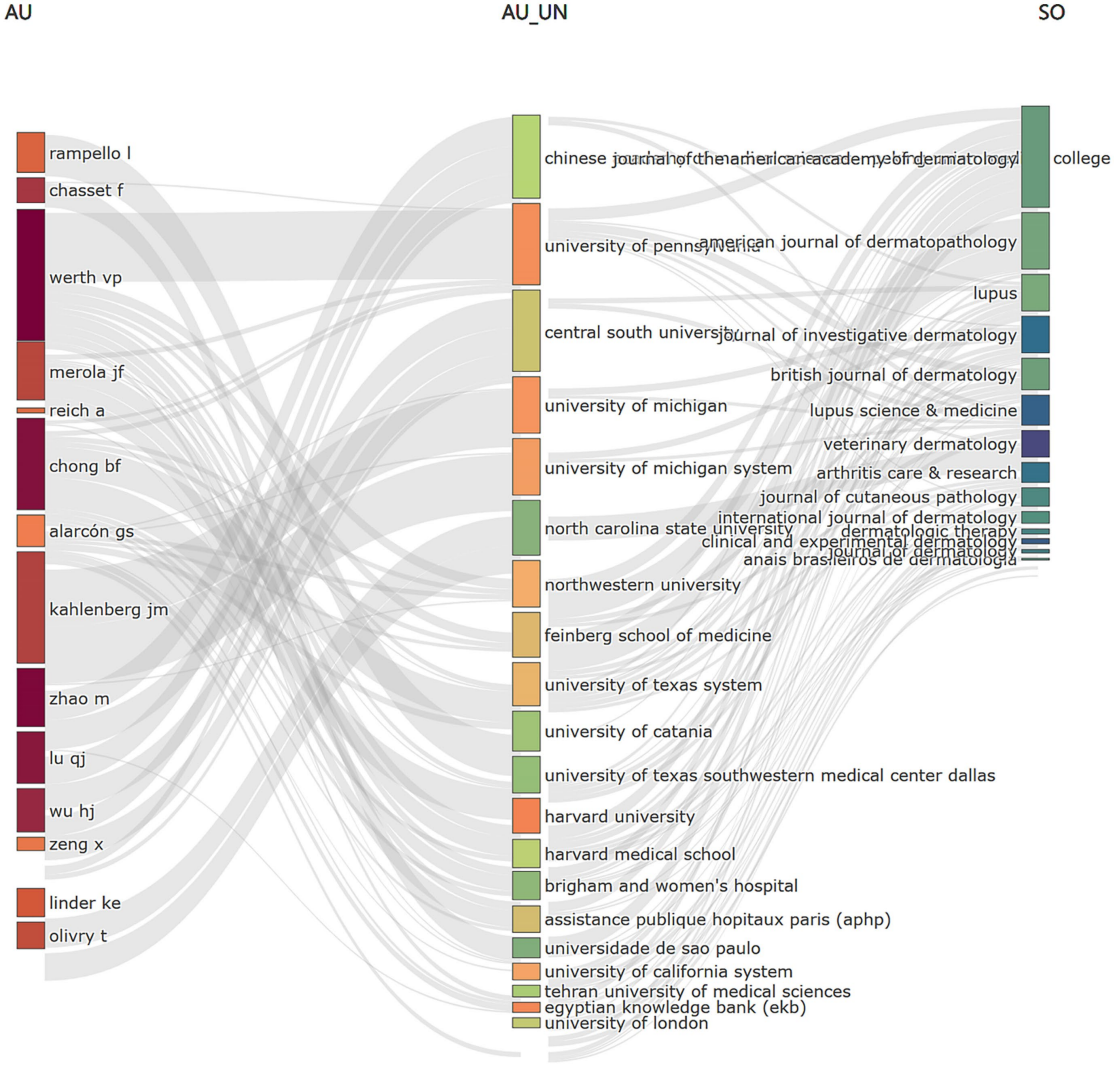
Three factor analysis. The rectangle diagram illustrates the main elements. The larger the rectangle, the stronger the relationship between the shown element and the others. AU, authors; AU_UN, institutions; SO, journals.

Figure 3 offers a comprehensive visualization of the interdisciplinary collaborations among authors, institutions, and journals within this research domain. Remarkably, the author WERTH VP has formed significant collaborations with leading institutions, such as the University of Pennsylvania, Northwestern University, and the Feinberg School of Medicine, among others. These collaborative efforts are prominently reflected in several key journals, including *Lupus*, *Journal of Investigative Dermatology*, and *Veterinary Dermatology*. Furthermore, the analysis reveals a marked concentration of research output in these select journals, underscoring their pivotal role in advancing the field. This pattern of collaboration and publication reflects broader trends within the discipline, highlighting the critical interconnections between institutions and journals.

### 3.3 Distribution of journals

**Figure 4:**
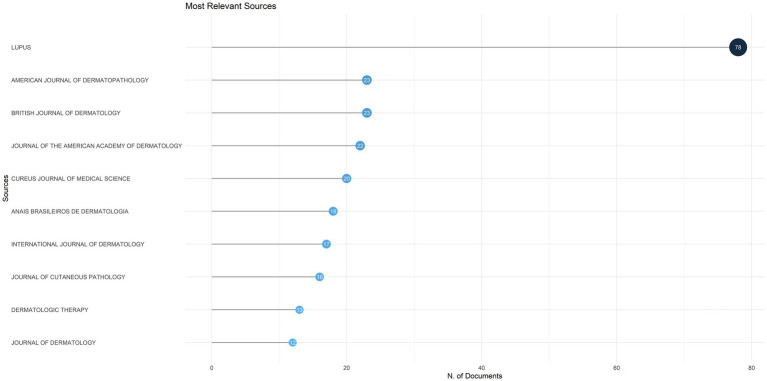
The top 10 journals by publication volume on discoid lupus erythematosus.

A total of 861 publications on DLE were published across 329 different journals. The top 10 journals by publication volume are listed in Figure 4. The journal with the highest publication volume was *Lupus* from England, with 78 publications, followed by the *American Journal of Dermatopathology* and *British Journal of Dermatology*, each contributing 23 publications.

### 3.4 Distribution of authors and collaboration network

The collected literature concerning DLE comprised contributions from a total of 4,149 authors, with the leading 13 authors ranked by their publication output displayed in Table 1. Significantly, the author with the most substantial publication output was WERTH VP from the United States, followed by ZHAO M and CHONG BF from China.

**Table 1:** The top 13 authors by publication volume on discoid lupus erythematosus.

Authors	Articles
Werth VP	24
Zhao M	11
Chong BF	10
Lu QJ	9
Tosti A	9
Wu HJ	9
Miteva M	8
Chasset F	7
Errichetti E	7
Kahlenberg JM	7
Merola JF	7
Olivry T	7
Wenzel J	7

**Figure 5:**
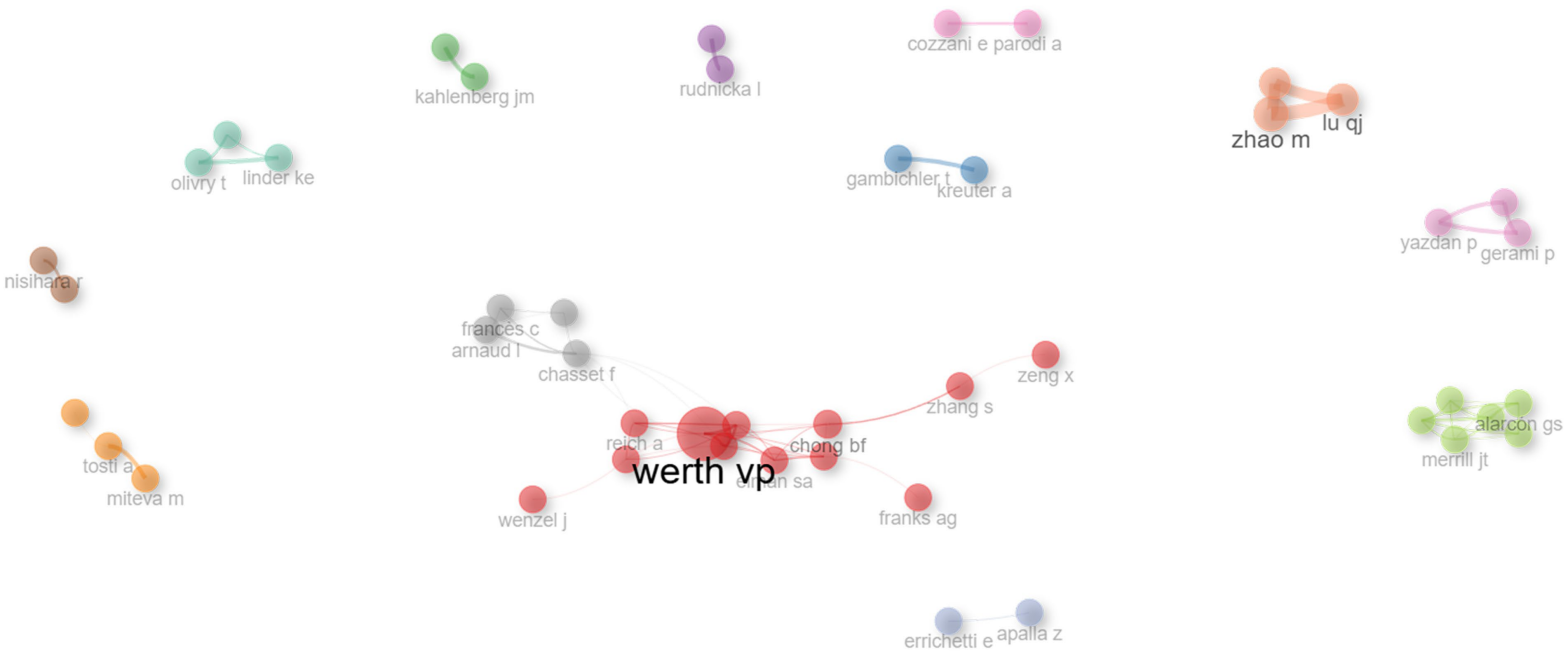
Author collaboration network. Nodes represent authors, with node size indicating publication count. Links reflect co-authorships, and link thickness corresponds to collaboration strength. Clusters highlight groups of closely collaborating researchers within the field.

The collaborative network among authors was categorized into 13 distinct clusters (Figure 5), where each color signifies a unique group of authors working together. The largest nodes, representing the most prolific contributors in this domain, include WERTH VP, CHONG BF, MEROLA JF, among others, reflecting their extensive publication records in this area of research.

### 3.5 Cooperation of countries and regions

**Figure 6:**
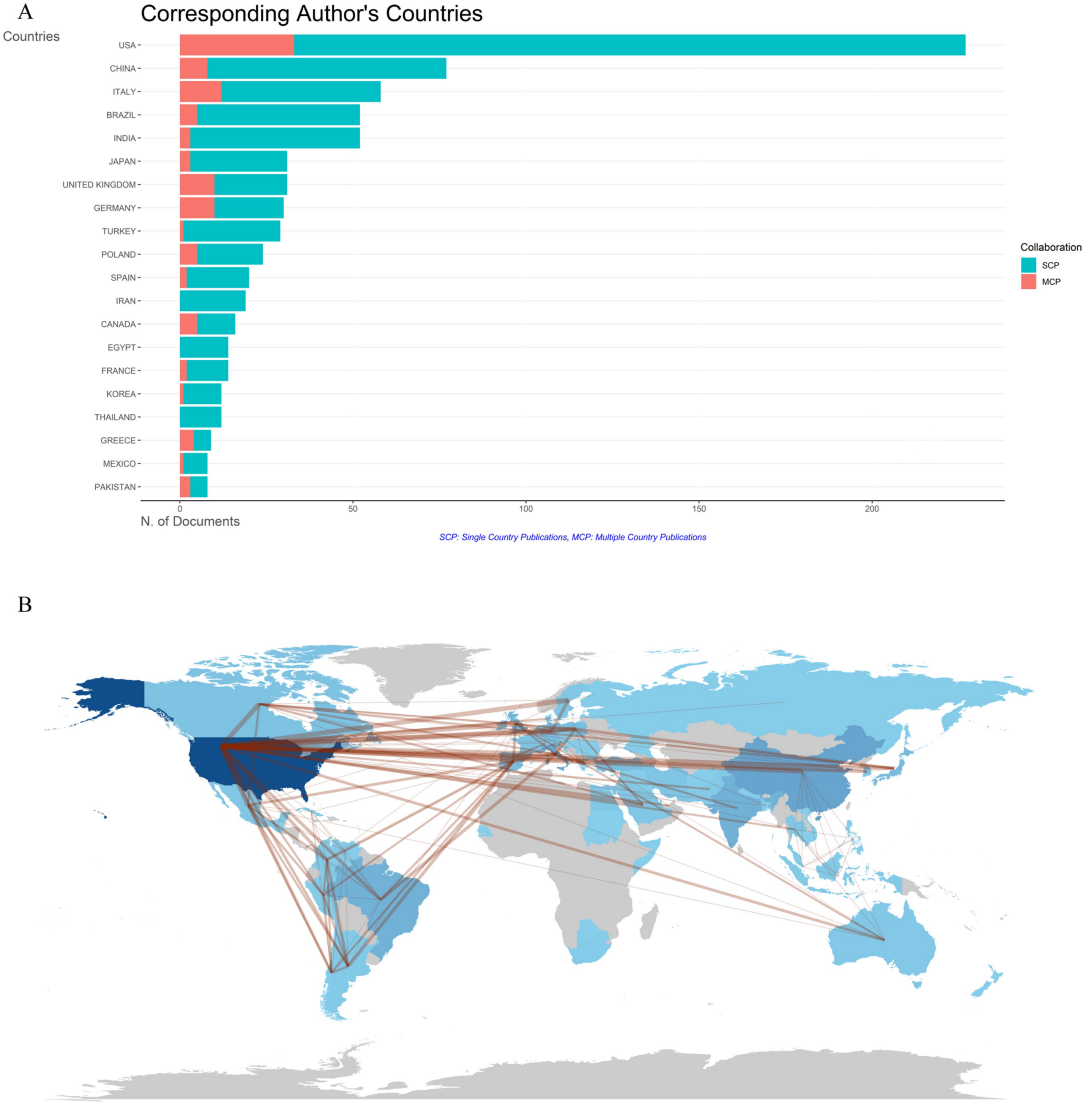
Countries/regions co-authorship analysis. **(A)** Histogram of cooperation in the top 20 productive countries/regions. The red section represents multiple country/region collaborative publications (MCP), and the green section defines single country/region publications (SCP). **(B)** Geographic maps in publication and collaboration of countries/regions.

Publications on DLE originated from 72 countries/regions. The bar chart in Figure 6A clearly illustrates that the top five countries with the highest levels of international collaboration are the United States, Italy, the United Kingdom, Germany, and China. Among them, the United States leads with 33 internationally co-authored publications, accounting for 14.5% of its total output. This trend highlights the growing importance of international cooperation in DLE research. Figure 6B depicts the cooperation patterns between different countries/regions, with dark blue representing higher publication volumes and stronger red indicating closer collaboration. Particularly, the United States not only has the most publications but also engages in the most extensive cooperation with other countries.

### 3.6 Most cited articles

Table 2 presents the 20 most frequently cited articles in DLE research. The article with the highest citation count, titled “*Oral potentially malignant disorders: A consensus report from an international seminar on nomenclature and classification, convened by the WHO Collaborating Centre for Oral Cancer*,” was authored by Warnakulasuriya et al. and published in *Oral Diseases* in 2021 (27).

**Table 2:** Top 20 most highly cited articles of discoid lupus erythematosus.

Article	Total citations
Warnakulasuriya et al. (27), Oral Dis	496
Thakor et al. (63), Nano Lett	375
Merrill et al. (64), Arthritis Rheum-US	289
Holland (65), Clin Rev Allerg Immu	261
Errichetti et al. (66), Dermatology Ther	205
Heckmann et al. (67), J Mol Biol	187
Holland (68), Hematol Oncol Clin N	178
Okon et al. (35), Best Pract Res Cl Rh	177
Sarkar et al. (69), Ann Rheum Dis	161
Frangou et al. (70), Ann Rheum Dis	160
Fallah et al. (71), Ann Oncol	142
Stefanato (72), Histopathology	134
Carrozzo et al. (73), Periodontol 2000	127
Min et al. (74), Exp Dermatol	125
Osio-Salido et al. (75), Lupus	117
Prencipe et al. (76), J Allergy Clin Immun	117
Braunstein et al. (77), Brit J Dermatol	114
Wenzel et al. (36), Nat Rev Rheumatol	114
Gill et al. (78), J Am Acad Dermatol	113
Hemminki et al. (79), J Autoimmun	110

### 3.7 Lotka’s and Bradford’s law

**Figure 7:**
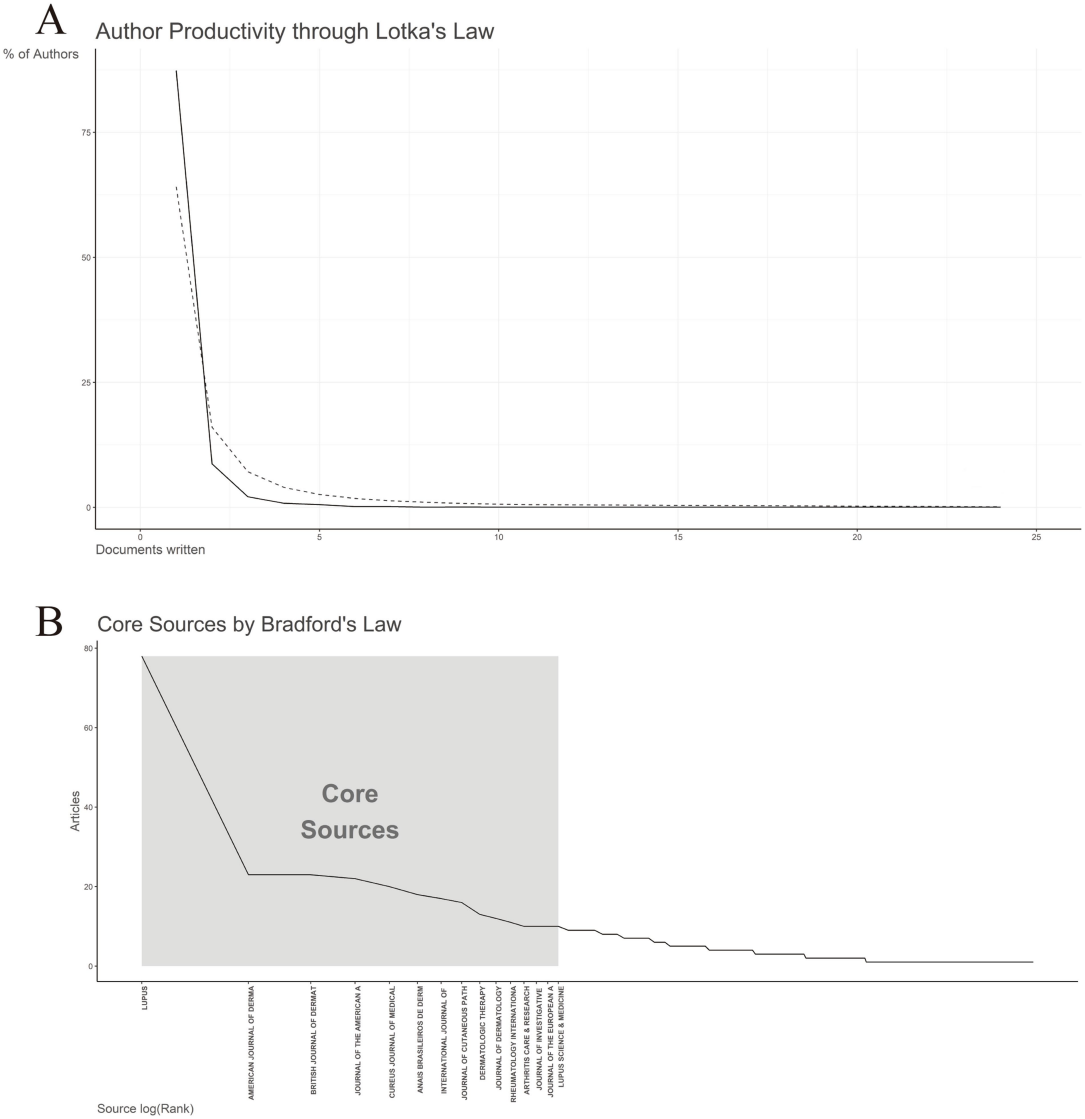
Lotka’s and Bradford’s law. **(A)** Author’s productivity analysis by Lotka’s Law. **(B)** Journals clustering through Bradford’s law.

Lotka’s law describes the distribution of author productivity across various scientific disciplines, typically indicating that a small proportion of authors is responsible for the majority of scholarly output, while a larger group contributes relatively few publications (28). An analysis of author productivity in line with Lotka’s law (Figure 7A) shows that only a small subset of authors has published 10 or more articles (*n* = 3, 0.07%), whereas the vast majority (*n* = 3,624, 87.35%) have authored just a single publication. Notably, one author stands out with a remarkable contribution of 24 articles.

Bradford’s law posits that journal citations are unevenly distributed across journals in any given subject area, with a small number of core journals accounting for a disproportionately large share of publications (29). As illustrated in Figure 7B, a few key journals, such as *Lupus* and *American Journal of Dermatopathology*, contributed approximately 60% of the total articles analyzed. These journals constitute the “core sources,” characterized by their high productivity and central role in disseminating research findings in the field. Beyond this core group, a set of secondary journals contributed a moderate number of publications, followed by a long tail of tertiary journals, each accounting for only a small fraction of the total output. This distribution underscores the concentration of knowledge in the field in a limited number of high-impact sources, aligning with Bradford’s theoretical framework. Identifying these journal zones (core, secondary, and tertiary) enhances researchers’ ability to target key publications and optimize literature searches in the field.

### 3.8 Co-cited references

**Figure 8:**
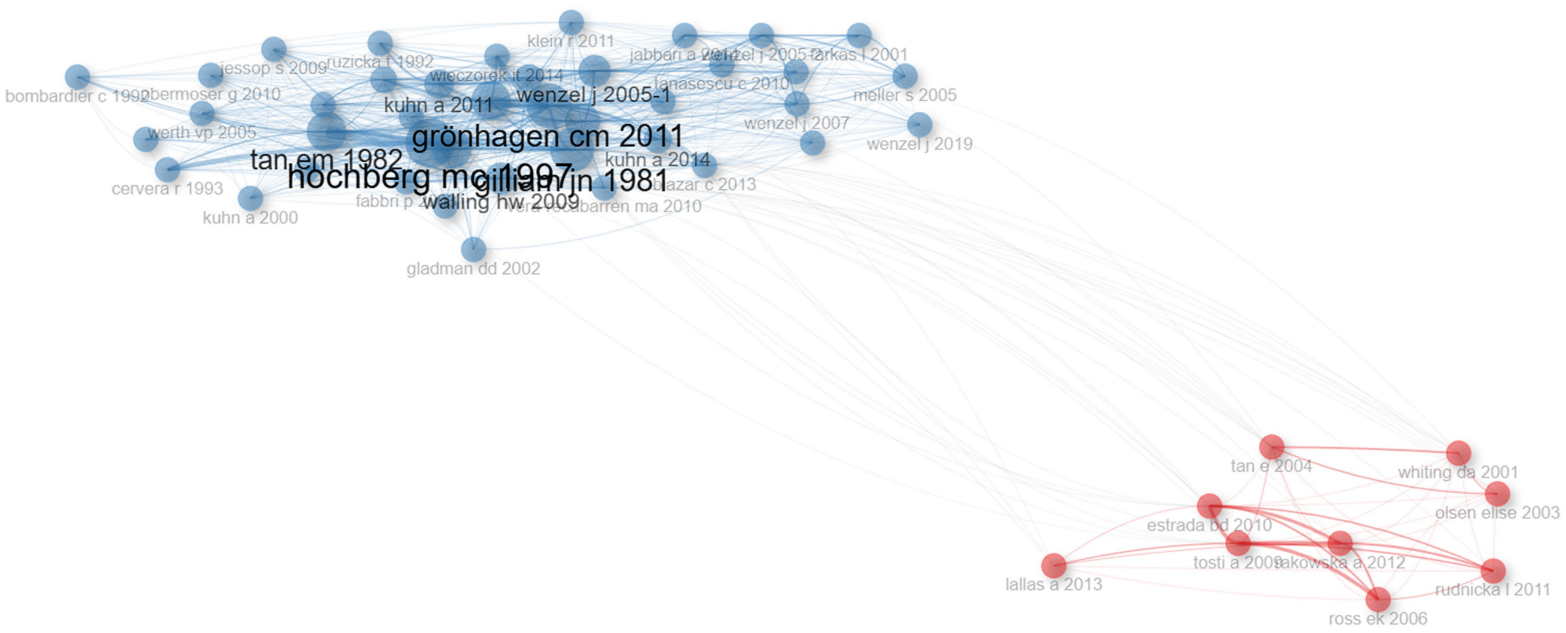
Co-citation network. Each node represents an article, and the connections between them reflect how often they are cited together. The size of the nodes corresponds to the number of citations, and the thickness of the lines indicates the strength of the co-citation relationship. The network helps to identify key studies and emerging research trends in the field.

Each of the co-cited references listed in Table 3 has been cited at least 40 times, with the most cited being *HOCHBERG MC, 1997, ARTHRITIS RHEUM* which has been co-cited over 100 times. A co-citation network analysis of the literature, as shown in Figure 8, identified two clusters, and the most cited article in the blue cluster, *Updating the American College of Rheumatology revised criteria for the classification of systemic lupus erythematosus*, was published in *Arthritis Rheumatology* in 1997 (30), is central to the understanding of SLE and has significantly influenced the classification criteria for lupus-related disorders. This article’s extensive citation indicates its foundational role in advancing diagnostic criteria for lupus. The most cited article in the red cluster, *Follicular red dots: a novel dermoscopic pattern observed in scalp discoid lupus erythematosus*, was published in *Archives of Dermatology* in 2009 (31). Its high citation count underscores its significant impact on DLE diagnosis, providing valuable insights that have since been widely adopted in clinical practice. These articles underscore the critical contributions of diagnostic criteria to the ongoing research and clinical management of DLE.

**Table 3:** Top 10 co-cited references in the field of discoid lupus erythematosus.

Cited reference	Citations
Hochberg (30), Arthritis Rheum	114
Tan et al. (80), Arthritis Rheum	81
Petri et al. (81), Arthritis Rheum-US	68
Gilliam et al. (82), J Am Acad Dermatol	61
Albrecht et al. (83), J Invest Dermatol	56
Grönhagen et al. (84), Brit J Dermatol	54
Walling et al. (85), Am J Clin Dermatol	53
Durosaro et al. (86), Arch Dermatol	52
Okon et al. (35), Best Pract Res Cl Rh	43
Kuhn et al. (87), J Autoimmun	40

### 3.9 Cluster analysis

**Figure 9:**
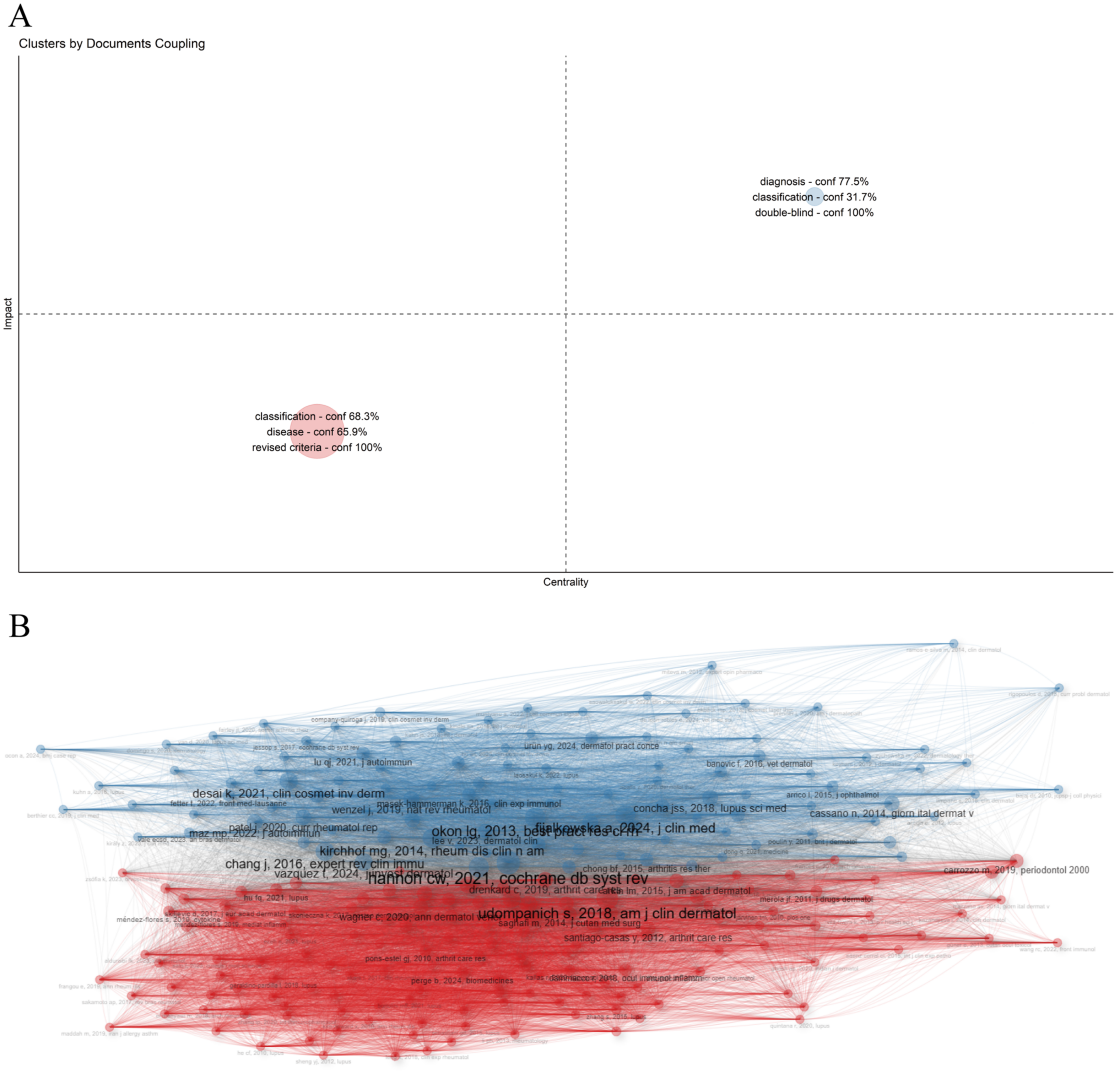
Cluster analysis. The network is divided into clusters, each representing a group of articles with similar co-citation patterns. Different colors distinguish the clusters, which highlight key research topics or trends in the field. **(A)** Cluster map. **(B)** Cluster by coupling network.

By analyzing the coupling relationships between literature or authors, such as mutual citation, collaboration, or shared focus, we can categorize them into distinct groups (32). As shown in Figure 9A, two primary clusters were identified, each characterized by distinct research foci and attributes. The first cluster is labeled “classification - conf 68.3% disease - conf 65.9% revised criteria - conf 100%” The second cluster is labeled “diagnosis - conf 77.5% classification - conf 31.7% double-blind - conf 100%,” conf (confidence) represents the degree to which a particular keyword is associated with a given cluster. The first cluster predominantly includes key studies such as *CHONG BF, 2012, BRIT J DERMATOL*, *WIECZOREK IT, 2014, JAMA DERMATOL* (33, 34), which are fundamental in refining lupus classification. The second cluster primarily features important works by *OKON LG, 2013, BEST PRACT RES CL RH*, *WENZEL J, 2019, NAT REV RHEUMATOL* (35, 36), contributing to advancements in diagnostic techniques and clinical trials (Figure 9B). These clusters emphasize the continued focus on refining DLE diagnosis and classification.

### 3.10 Identification and analysis of keywords

**Figure 10:**
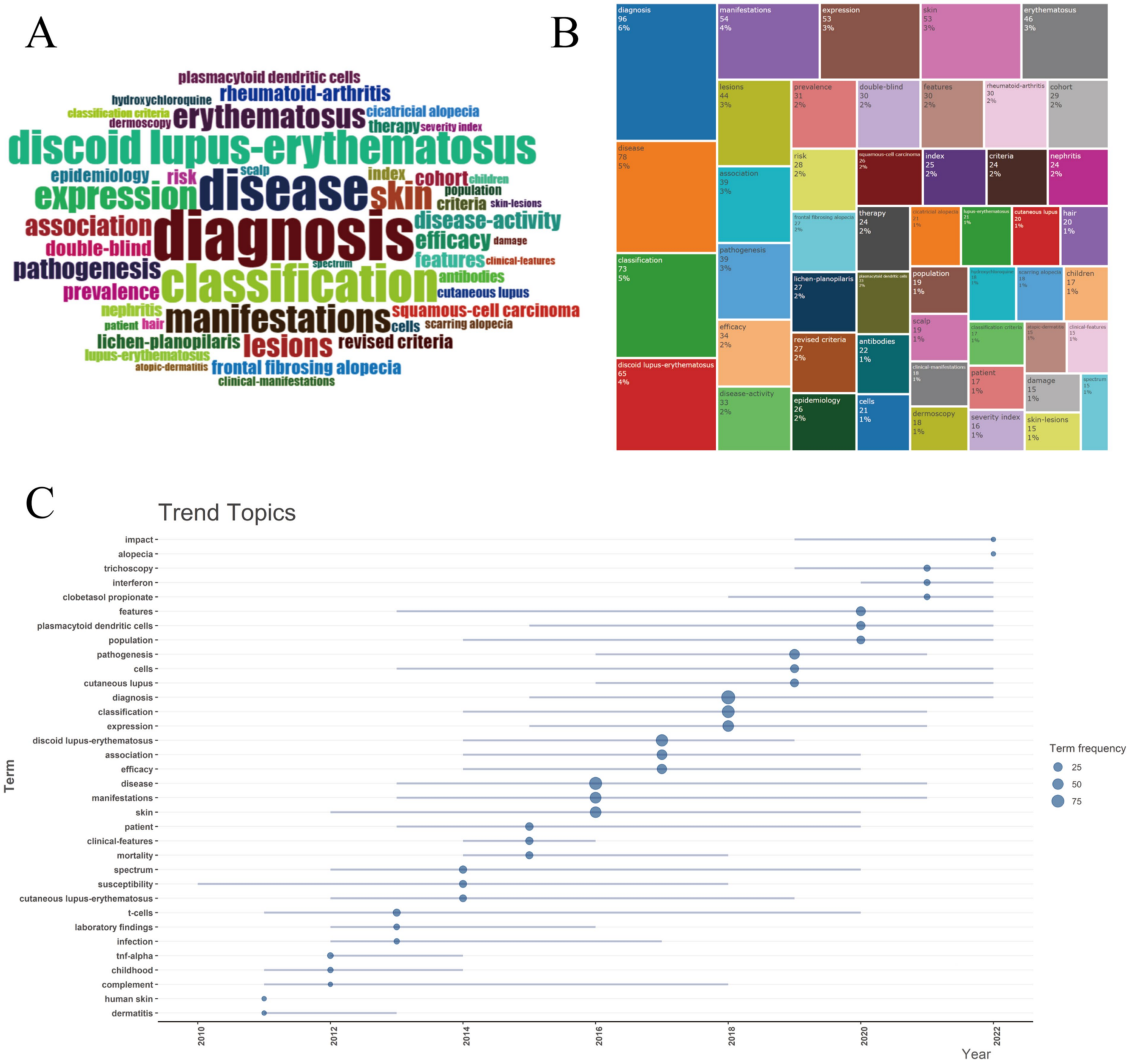
Keywords and trend topics. **(A)** Word cloud. **(B)** Tree map. **(C)** Trend topics. Circle size corresponds to keyword frequency counts (scale: 25 = 25 occurrences, 50 = 50 occurrences, 75 = 75 occurrences), and line length quantifies temporal span.

Keywords in the retrieved articles were analyzed to assess the key topics within the field of DLE. The word cloud in Figure 10A and the tree diagram in Figure 10B illustrate the most frequently occurring keywords. Markedly the top three keywords—diagnosis disease and classification—highlight the primary focus areas of research in this domain. The changing trends of hot keywords related to DLE over the past 15 years are depicted in Figure 10C. Conspicuously t-cells was the most persistent keyword appearing consistently from 2011 to 2020. In recent years emerging keywords such as trichoscopy interferon and clobetasol propionate have gained prominence highlighting treatment and diagnosis as the forefront of DLE research.

**Figure 11:**
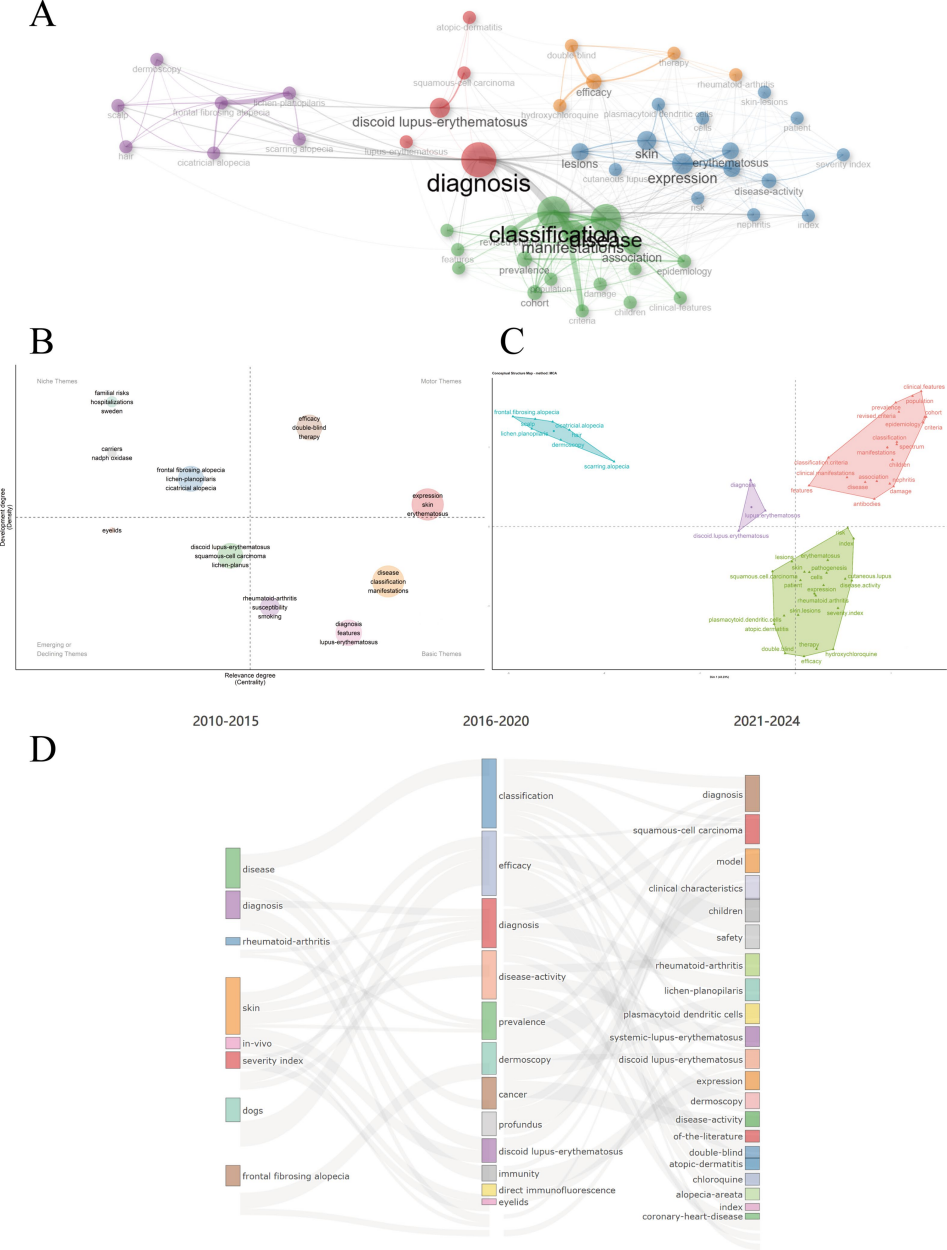
Thematic structure and evolution. **(A)** Co-occurrence network. **(B)** Thematic map. The x-axis represents the center and measures the relevance between topics, while the y-axis indicates the density. A higher density signifies a more mature area of research. **(C)** Factor analysis. Each factor represents a key theme in the research, helping to identify major trends and simplify complex data. **(D)** Thematic evolution.

A comprehensive overview of DLE research themes, their structural relationships, and temporal evolution is provided in Figure 11. The co-occurrence network (Figure 11A) reveals 49 keywords grouped into five distinct clusters, highlighting key topics such as diagnosis, expression, classification, and manifestations. The thematic map (Figure 11B) categorizes research topics into four quadrants based on their relevance and stage of development. Motor themes (upper-right quadrant), such as therapy, efficacy, and double-blind studies, exhibit higher centrality and density values. Basic themes (lower-right quadrant), including disease, classification, and manifestations, are fundamental to understanding DLE. Emerging or decline themes (lower-left quadrant), such as squamous-cell carcinoma and lichen planus, are still under development. Niche themes (upper-left quadrant), including frontal fibrosing alopecia and cicatricial alopecia, are well-developed but remain at the periphery of the research domain. Factor analysis (Figure 11C) revealed four main clusters of research: diagnostic methods (discoid lupus erythematosus, diagnosis), clinical features and classification (classification, manifestations), basic research on molecular mechanisms (expression, therapy, pathogenesis), and specific scalp manifestations (frontal fibrosing alopecia, hair). These clusters emphasize that advancements in diagnosis and clinical classification remain central to understanding DLE. The thematic evolution (Figure 11D) reveals distinct temporal phases in DLE research: early studies (2010–2015) primarily focused on clinical descriptions and diagnosis, followed by a growing emphasis on classification and efficacy (2016–2020). In recent years (2021–2024), there has been rapid growth in niche topics, advanced therapies, and rare manifestations. This trend reflects the expanding scope of DLE research, encompassing both fundamental studies and clinical applications.

## 4 Discussion

Discoid lupus erythematosus (DLE) presents both a medical challenge due to its physical manifestations and a significant psychosocial burden. The conspicuous facial lesions, in particular, can cause considerable psychological distress and reduce self-esteem, especially among young women who may be more concerned about their appearance (37). DLE recognized as a cutaneous manifestation of systemic lupus erythematosus (SLE) (38). It remains relatively underrepresented in the broader lupus literature compared to systemic lupus erythematosus (SLE), leading to notable gaps in our understanding of its pathogenesis, management, and outcomes. This bibliometric study aimed to address these gaps by comprehensively analyzing DLE-related publications from 2010 to 2024, focusing on scientific output trends, collaboration patterns, core journals, key authors, and emerging research fronts. The findings yield valuable insights into the developmental trajectory of DLE scholarship and provide a roadmap for future investigations.

### 4.1 Overall growth trends in DLE research

A total of 861 publications were retrieved from the Web of Science Core Collection (WoSCC) database, covering the period from January 2010 to November 2024. Our analysis reveals a fluctuating yet generally increasing annual output of DLE-related articles, with a more pronounced rise observed after 2018. The observed acceleration in DLE-related publications has coincided with the increasing clinical adoption of biologic therapies, such as anifrolumab, which were initially developed for SLE but also have shown efficacy in refractory cutaneous diseases, including DLE. Emerging case reports and clinical studies highlighting the novel application of these biologics may have contributed to renewed research interest in DLE therapies (39, 40). This upward trend indicates a growing scholarly interest in DLE, consistent with the broader global emphasis on autoimmune and autoinflammatory conditions (41, 42). However, the total number of citations demonstrated a downward trajectory from 2010 to 2024. This paradox—rising publication counts accompanied by decreasing average citations—may reflect several factors. Firstly, the proliferation of specialized journals and open-access platforms has increased the overall volume of output but resulted in a dispersion of citations across a broader range of venues (43). Additionally, DLE research often intersects with SLE and other autoimmune diseases, which often results in citation practices favoring high-impact journals focused on broader autoimmune research rather than those specifically dedicated to DLE. Moreover, articles published toward the end of the study period (e.g., 2022–2024) have had less time to accumulate citations, resulting in a temporal lag in citation metrics. Overall, while the growing quantity of DLE-related research reflects increased awareness, the declining citation density presents a potential challenge in consolidating findings, bridging research domains, and directing attention toward highly impactful works.

### 4.2 Core journals and application of Bradford’s law

Bradford’s law posits that literature within a specialized domain is primarily concentrated in a limited number of core journals, with productivity declining sharply in secondary and tertiary sources (44). In our study, *Lupus* (England) emerged as the highest-volume journal, publishing 78 articles, followed by the *American Journal of Dermatopathology* with 23 articles. This distribution exemplifies Bradford’s law, whereby key journals in a given area account for a disproportionately large share of publications—approximately 60% of the total—as depicted in Figure 7B. The concentration of DLE literature in these leading journals highlights the domain specialization inherent in the field, as journals like *Lupus* and *American Journal of Dermatopathology* serve as essential conduits for disseminating findings related to lupus pathogenesis, immunology, and dermatologic manifestations. Additionally, authors preferentially submit high-quality work to these journals due to their long-standing reputations, established editorial boards, and recognized expertise in lupus research. However, while this specialization fosters in-depth coverage, it may also limit cross-disciplinary integration, making it more challenging for fields such as immunology, rheumatology, and dermatology to converge around DLE-focused studies. Therefore, expanding DLE research dissemination across multidisciplinary fields remains critical, especially as emerging therapeutic strategies increasingly intersect with rheumatology, immunology, and genetics (45, 46).

### 4.3 Leading authors and collaboration networks

Consistent with Lotka’s law, our study revealed that a small number of authors produced the majority of publications (47), while the vast majority authored only a single article (87.35%). This pattern is particularly evident in a condition such as DLE, which remains relatively niche compared to more common dermatological or autoimmune disorders (e.g., rheumatoid arthritis). A few highly engaged experts such as WERTH VP and CHONG BF lead the field through highly cited publications.

Bibliometric mapping uncovered 13 distinct author clusters (Figure 5), reflecting cohesive research collaborations. The structure of these author networks indicates that certain clusters are centered around shared institutional affiliations (e.g., University of Pennsylvania, Northwestern University), while others are driven by thematic overlaps, such as immunological aspects of lupus. Identifying these clusters allows new and aspiring investigators to pinpoint established teams, thereby fostering meaningful partnerships and multi-center collaborations. Strong collaborative patterns are often correlated with increased scientific impact (48). Indeed, multi-institutional and international collaborations not only amplify sample sizes and geographic diversity but also integrate interdisciplinary expertise—from immunologists and rheumatologists to dermatologists and geneticists. Given the complexity of lupus pathogenesis, robust collaborations are essential for advancing personalized therapies, refining diagnostic criteria, and improving patient quality of life.

### 4.4 Countries and international cooperation

As illustrated in Figures 6A,B, the United States leads in both publication volume and collaborative linkages, followed by Italy, the United Kingdom, Germany, and China. This geopolitical distribution aligns with broader trends in lupus and immunology research, where high-income countries with well-established research infrastructures dominate the field. Especially, China’s emergence among the top five contributors highlights its growing focus on autoimmune diseases, driven by substantial governmental funding, rapid economic development, and a significant increase in academic output (49). Autoimmune diseases demonstrate varying prevalence and distinct clinical presentations across regions, shaped by genetic and environmental factors (50, 51). Strengthening international partnerships can accelerate the identification of novel biomarkers, epigenetic mechanisms, and advanced therapeutics to address diverse disease phenotypes.

### 4.5 Keyword and cluster analysis

Keyword analysis revealed shifting research emphases in DLE over time. Early studies (2010–2015) focused on basic clinical descriptions such as disease and diagnosis alongside broad immunopathological processes. Between 2016 and 2020 the focus expanded to include classification efficacy and therapy. Recent years have witnessed a rise in niche topics including trichoscopy interferon and clobetasol propionate reflecting advancements in dermatologic diagnostic methods for scalp lesions and novel immunotherapeutic strategies targeting the interferon pathway (52, 53). These trends highlight significant strides in specialized areas like advanced diagnostic techniques (e.g., dermoscopy trichoscopy) and immunopathological research including cytokine and chemokine profiling (54, 55). While clobetasol propionate a potent topical corticosteroid is a novel treatment for DLE (56). Its efficacy stems from its strong anti-inflammatory and immunosuppressive properties which help reduce interface dermatitis and prevent irreversible scarring clinical studies have demonstrated that in patients with DLE topical 0.05% clobetasol propionate ointment offers significantly greater therapeutic benefits than 0.1% tacrolimus ointment (57). To improve its safety profile novel delivery systems are under investigation. For instance clobetasol propionate–loaded nanosponge hydrogels enable controlled drug release and markedly reduce adverse effects such as skin atrophy (58). However these innovations remain in preclinical or early-phase development with no registered clinical trials specifically targeting DLE to date.

Cluster analysis identified distinct thematic groupings in DLE research. The first cluster centered on classification and revised diagnostic criteria, highlighting ongoing efforts to refine disease definitions and outcomes, particularly given the overlap between DLE and SLE. The second cluster focused on diagnostic methods and double-blind studies, aiming to differentiate DLE from conditions such as psoriasis, lichen planus, and other forms of cutaneous lupus. These studies emphasize the growing demand for evidence-based interventions. The others showcased scalp-specific presentations of DLE, including frontal fibrosing alopecia, cicatricial alopecia, and associated dermoscopy patterns. Typical dermoscopic findings in DLE include white structureless areas, arborizing vessels, white scales, and follicular keratin plugs—features that help distinguish it from other alopecias (59, 60). Dermoscopy can also reveal characteristic features in non-scalp regions, including the face, trunk, and limbs, where keratotic follicular plugs, peripilar white halos, and surface scaling are frequently observed. Recognizing these patterns is essential for early diagnosis and timely intervention in DLE (61).

### 4.6 Most cited articles and co-citation analysis

Interestingly, the most cited article, authored by Warnakulasuriya in 2021 (27), focuses on oral potentially malignant disorders. While this topic may initially seem tangential to DLE, it highlights the intersection between mucocutaneous lesions and the broader field of oral diseases. Consistent co-citations indicate that these works provide foundational conceptual or methodological contributions, such as defining DLE, advancing imaging techniques for diagnosis, or refining classification criteria. The strong co-citation of the 1997 American College of Rheumatology criteria (30) reflects their enduring relevance. These criteria align with ongoing efforts to refine lupus classification, aiming to distinguish systemic and discoid manifestations and guide clinical trial enrollment effectively.

### 4.7 Clinical and research significance

DLE presents diagnostic and therapeutic challenges due to its variable clinical spectrum, overlap with SLE, and distinct subtypes (62). This bibliometric study highlights diagnosis and classification as crucial themes, emphasizing the need for refined diagnostic algorithms to improve early detection, reduce misdiagnosis, and optimize interventions. Thematic evolution reveals a growing focus on therapy and efficacy, while hydroxychloroquine remain standard treatments, future strategies are likely to incorporate novel immunomodulators in combination regimens.

### 4.8 Strengths and limitations

This study offers several strengths. The analysis spans from 2010 to 2024, capturing the recent surge in DLE research and providing a comprehensive overview of current priorities and collaboration patterns. It incorporates diverse metrics such as co-authorship, co-citation, keywords co-occurrence, thematic mapping, and factor analyses. Additionally, identifying leading authors, core journals, and key topics establishes a structured knowledge base that clinicians and researchers can use to guide new projects, prioritize funding, and advance translational research.

However, some limitations should be noted. Restricting data retrieval to WoSCC may exclude relevant articles indexed in PubMed, Scopus, or other databases, potentially underrepresenting the global literature. The inclusion of only English-language articles introduces a bias against research from non-English-speaking regions. Finally, bibliometric measures may undervalue recent high-quality studies due to citation lag and do not account for the sentiment of citations (positive, neutral, or negative).

### 4.9 Future directions

Refining diagnostic criteria for DLE remains a critical priority. Advanced molecular and genetic studies, such as single-cell RNA sequencing, can provide insights into disease heterogeneity and identify personalized therapeutic targets. Large-scale studies are needed to evaluate the efficacy of biologics and small-molecule inhibitors while exploring combination therapies with conventional agents like hydroxychloroquine. Expanding collaborations with underrepresented regions could improve the global applicability of findings and uncover unique genetic or environmental factors influencing disease expression. Furthermore, leveraging AI and machine learning with clinical datasets and imaging repositories has the potential to transform diagnostics, refine classification systems, and accelerate drug discovery.

## 5 Conclusion

This bibliometric study highlights the increasing scholarly focus on discoid lupus erythematosus (DLE) from 2010 to 2024, despite relatively low average citation rates. Key journals, such as *Lupus*, dominate publication output, and the United States lead in research collaborations. The initial emphasis on diagnostic criteria and disease classification has progressively shifted toward advanced therapies and immunological mechanisms. Expanding collaborations and conducting deeper molecular investigations are essential for refining DLE management and improving patient outcomes.

## Data Availability

The original contributions presented in the study are included in the article/supplementary material, further inquiries can be directed to the corresponding author.
